# Outcomes of Orthopaedic Trauma Services at a UK Major Trauma Centre During a National Lockdown and Pandemic: The Need for Continuing the Provision of Services

**DOI:** 10.7759/cureus.11056

**Published:** 2020-10-20

**Authors:** Richard L Donovan, Thomas Tilston, Rhiannon Frostick, Tim Chesser

**Affiliations:** 1 Trauma and Orthopaedic Surgery, North Bristol National Health Service (NHS) Trust, Bristol, GBR

**Keywords:** covid-19, orthopaedic trauma, trauma, trauma centers, pandemics, public health, hip fractures, emergency surgery, fractures, orthopaedics surgery

## Abstract

Aim

To review the trauma operating workload, theatre time and outcomes at a time of national lockdown at the beginning of the coronavirus disease 2019 (COVID-19) pandemic, comparing it with a year prior.

Methods

A retrospective case-control study was performed in a single Level 1 Major Trauma Centre (MTC) in the UK. Inclusion criteria were all patients undergoing operative intervention for an emergency or urgent trauma admission within our Trauma and Orthopaedics department. Data collected included anatomical area of injury, cause of injury, operative procedure, type of anaesthesia, total theatre time, complications, and mortality at 30 days.

Results

A total of 159 operations were performed on 142 patients in April 2019, and 110 operations on 106 patients in April 2020 (time of national lockdown). There was a 30% decrease due to reduced numbers of road traffic accidents and sport-related injuries. The number of hip fractures and those injuring themselves from less than 2m height remained the same. Operative total theatre time increased by a mean of 14 minutes, and complications and mortality were not significantly changed. The incidence of COVID in the patients tested was 8.5%, which matched the population incidence at the time.

Conclusions

Orthopaedic trauma services need to be provided during a national lockdown. There was no decrease in the volume of patients sustaining falls, which includes hip fractures. Mean operating time only increases by 14 minutes with the wearing of PPE. This should be part of future planning of any pandemics or national lockdowns.

## Introduction

Severe acute respiratory syndrome coronavirus 2 (SARS-CoV-2) was first reported in late 2019 in Wuhan, China, and within three months had spread to most countries in the world. On March 11, 2020, the World Health Organisation declared coronavirus disease 2019 (COVID-19) a pandemic [1].

On March 23, 2020, the UK government introduced a national lockdown, limiting all non-essential work, non-essential travel, and all social and sports gatherings. Hospitals cancelled all non-urgent surgery and adapted to receive multiple patients suffering from COVID-19 during the initial surge of infections in the first wave of the pandemic. Orthopaedic trauma departments continued to function, though with limited face to face consultations and advocated conservative treatment where possible [2]. Operating was undertaken following national guidelines for safety, using full personal protective equipment (PPE), for every case with aerosol-generating procedures, which included the significant majority of orthopaedic trauma cases [2-4]. Concern was raised of high mortality rates in patients undergoing orthopaedic trauma surgery from initial reports from China and Italy [5].

This study aimed to review the trauma operating workload, theatre time and outcomes, in April 2020, at a time of national lockdown and the beginning of the pandemic, comparing it with a year prior, April 2019.

## Materials and methods

A retrospective case-control study was performed in a single Level 1 Major Trauma Centre (MTC) in the UK. Inclusion criteria were all patients undergoing operative intervention for an emergency or urgent trauma admission within the Trauma and Orthopaedics department. Patients undergoing non-operative management of their injuries were excluded. All patients included were aged 16 or over. The unit studied looked after patients with limb and pelvic and acetabular injuries. Head, neck, spine, chest, and hand injuries were managed by other speciality departments within the same MTC.

Consecutive data were retrospectively collected for the period of April 01 to April 30, 2019 (control group), and April 01 to April 30, 2020 (case group). Data parameters collected included: date of admission, date of surgery, hospital number, date of birth, age (on the day of surgery), gender, diagnosis, anatomical area of injury, cause of injury, operative procedure, type of anaesthesia, total theatre time, lead anaesthetist grade, level of most senior surgeon present, operating surgeon grade, complications, and mortality.

Anatomical area of injury was sub-categorised into pelvis/acetabulum, hip, femur, knee, tib-fib, ankle, foot, clavicle, shoulder, arm, elbow, forearm, and wrist. Cause of injury was sub-categorised into road traffic accident, jump/fall from height (>2 metres), crush injury, fall from bicycle or horse, low energy fall/trauma, sports/recreational, fight/assault, other, and unknown. Time in theatre per operation is defined from the moment the patient enters the operating theatre to be anaesthetised, to the moment they leave the operating theatre. Complications within 30 days of surgery were collated and categorised into: wound complications, vascular injury, nerve injury, thromboembolic, anaemia requiring transfusion (post-op), cardiorespiratory, renal, and other. The 30-day mortality (all causes) data from the time of admission was gathered, along with available COVID swab data for the 2020 cohort.

Data parameters listed above were collected retrospectively by three researchers (RD, TT, RF). Any disputes were settled through consensus. Data sources included: admission clerking notes, ward round notes, operation notes, anaesthetic charts, radiographs, and theatre data. Institutional approval was obtained prior to commencing data collection.

To analyse the effect between case (April 2020) and control (April 2019) groups, odds ratios (OR) were calculated using MedCalc for Windows, version 15.0 (MedCalc Software, Ostend, Belgium). Statistics were expressed with alpha values of 0.05 and 95% confidence intervals. To compare the difference between time spent in theatre, the Mann-Whitney test was utilised, given that the data was non-parametric.

## Results

Participant demographics

Retrospective consecutive data was collected from 248 patients undergoing emergency or urgent Trauma and Orthopaedic surgery in North Bristol National Health Service (NHS) Trust, a UK Level 1 MTC. A total of 159 operations were performed on 142 patients between April 01 to April 30, 2019, and 110 operations on 106 patients between April 01 to April 30, 2020.

In April 2019, the 142 patients sustained a total of 165 injuries requiring operative intervention, of whom 11 patients underwent two operations and three patients underwent three operations. In April 2020, the 106 patients had sustained 124 injuries requiring operative intervention, and four patients underwent two operations.

Full datasets were available except for the time in theatre for six operations in April 2019 and eight operations in 2020. One patient died before surgery in April 2019. Table 1 shows the patient demographics. There was a 25% reduction in the number of patients requiring emergency or urgent operations and a 30% reduction in the number of operations performed when comparing April 2020 to April 2019. There was no significant difference in the volume of patients with falls less than 2m (including hip fractures) and polytrauma.

**Table 1:** Patient demographics

	April 2019	April 2020
Number of patients (n)	142	106
Gender, n (%)		
Male	71/142 (50%)	44/106 (42%)
Female	71/142 (50%)	62/106 (58%)
Age (y)		
Median (IQR)	60 (40 – 83)	71 (46 - 83)
Number of operations (n)		
Total	159	110
Mean per day	5.3	3.7
Polytrauma (n)	6/142	5/106
Open fracture (n)	15/142	8/106
Prosthetic joint infection (n)	3/142	2/106
Septic arthritis (n)	3/142	2/106
Pathological fracture (n)	3/142	2/106

Mechanism of injury, and anatomical area of injury requiring surgery

In April 2019, the most prevalent mechanisms of injury were falling from standing height (or less), road traffic accidents, and ‘other’ (13 infections, three metalwork complications, one carpal tunnel, one laceration). In April 2020, the most prevalent were falling from standing height (or less), falling from a horse or bicycle, and 'other' (four infections, two lacerations). There was a significant increase in the proportion of patients sustaining low energy trauma and falls from bicycles/horses, and a significant decrease in sports/recreational-related injuries. In April 2019, the 142 patients had a total of 165 injuries, and in April 2020, the 106 patients had sustained 124 injuries. There were no significant variations between individual anatomical areas of injury. The full breakdown of the mechanism of injury results is illustrated in Table 2.

**Table 2:** Comparison of mechanisms of injury

Mechanism of Injury	April 2019		April 2020		Odds Ratio (95% CI)	p-value
	n	%	n	%		
Road traffic accident (RTA)	20/142	14.1	7/106	6.6	0.43 (0.18, 1.06)	0.067
Jump/fall from height (>2 metres)	5/142	3.5	5/106	4.7	1.36 (0.38, 4.81)	0.637
Crush injury	3/142	2.1	4/106	3.8	1.82 (0.40, 8.30)	0.441
Fall from bicycle/horse	2/142	1.4	7/106	6.6	4.95 (1.01, 24.33)	0.049
Low energy fall/trauma	78/142	51.4	75/106	65.1	1.98 (1.16, 3.38)	0.012
Sports/recreational	14/142	9.9	1/106	0.9	0.09 (0.01, 0.67)	0.019
Fight/assault	1/142	0.7	0/106	0.0	0.44 (0.02, 10.98)	0.619
Other	18/142	12.7	6/106	5.7	0.41 (0.16, 1.08)	0.072
Unknown	1/142	0.7	1/106	0.9	1.34 (0.08, 21.72)	0.836

Mode of anaesthesia

In April 2020, several changes to the chosen modes of anaesthesia were observed. There was a significant increase in the use of spinal anaesthesia from 1% to 32% of cases. Table 3 displays the data for all modes of anaesthesia in April 2019 and April 2020.

**Table 3:** Comparison of modes of anaesthesia per operation

Mode of Anaesthesia per Operation	April 2019		April 2020		Odds Ratio (95% CI)	p-value
	n	%	n	%		
General anaesthesia (GA) only	93/158	58.9	42/110	38.2	0.43 (0.26, 0.71)	0.001
Spinal anaesthesia only	0/158	0.0	23/110	20.9	85.14 (5.11, 1418.83)	0.002
Regional anaesthesia only	2/158	1.3	4/110	3.6	2.94 (0.53, 16.36)	0.217
Local anaesthesia (LA) only	4/158	2.5	1/110	0.9	0.35 (0.04, 3.20)	0.355
GA + spinal	2/158	1.3	0/110	0.0	0.28 (0.01, 5.96)	0.417
GA + regional	56/158	35.4	26/110	23.6	0.56 (0.33, 0.97)	0.040
Spinal + regional	0/158	0.0	12/110	10.9	40.23 (2.36, 687.11)	0.011
Sedation only	0/158	0.0	2/110	1.8	7.30 (0.35, 153.65)	0.201
None	1/158	0.6	0/110	0.0	0.48 (0.02, 11.77)	0.650

Time in theatre per operation

There was a statistically significant increase in both the median and mean total theatre time per operation (14 minutes) in April 2020 compared with April 2019 (p=0.039, U=6808, Z=-1.764), as demonstrated in Table 4.

**Table 4:** Comparison of time in theatre per operation

Time in Theatre per Operation	April 2019	April 2020
Sample size	152	103
Median (IQR), minutes	165 (93 – 183)	172 (119 – 178)
Mean, minutes	130	144

Grades of staff present intra-operatively

In 2019, there was already almost universal Consultant anaesthetic presence in orthopaedic trauma theatres, which continued in April 2020. A significant increase in the Consultant surgeon presence in theatre was demonstrated (p=0.049) which was matched by a significant decrease in the number of cases where a registrar was the most senior surgeon present (p=0.049).

There was no significant change in the prevalence of the operating surgeon being a Consultant when comparing April 2020 to April 2019. However, it was observed that there was a significant increase in the prevalence of the operating surgeon being a Registrar (p=0.035). This appeared to be at the expense of Core Trainee/Senior House Officers, as the data demonstrated a significant decrease in their prevalence as the operating surgeon in April 2020 compared to April 2019 (p=0.008). Table 5 shows the full breakdown of the grades of staff present intra-operatively.

**Table 5:** Comparison of staff presence intra-operatively

Staff Presence per Operation	April 2019		April 2020		Odds Ratio (95% CI)	p-value
	n	%	n	%		
Grade of lead anaesthetist						
Consultant	153/158	96.8	109/110	99.1	3.56 (0.41, 30.92)	0.249
N/a (local anaesthesia)	5/158	3.2	1/110	0.9	0.28 (0.03, 2.44)	0.249
Grade of most senior surgeon						
Consultant	121/158	76.6	95/110	86.4	1.94 (1.00, 3.74)	0.049
Registrar	37/158	23.4	15/110	13.6	0.52 (0.27, 1.00)	0.049
Grade of operating surgeon						
Consultant	47/158	29.7	30/110	27.3	0.89 (0.52, 1.52)	0.660
Registrar	92/158	58.2	78/110	70.9	1.75 (1.04, 2.94)	0.035
Core Trainee/Senior House Officer	19/158	12.0	2/110	1.8	0.14 (0.03, 0.59)	0.008

Complication rates

In April 2019, 34 postoperative complications were noted in 27 patients; and in April 2020, 33 postoperative complications were noted in 24 patients. Overall, a mild increase in the complication rate was observed between April 2019 and April 2020, but no significant difference was demonstrated (OR 1.29; CI: 0.69, 2.41; p=0.417). Sub-classification analysis of complications unveiled a significant increase in renal post-operative complications only. A full list of complications is provided in Appendix 1.

30-day mortality rates

Overall, an increase in the 30-day mortality rate was observed between April 2019 (six patients) and April 2020 (eight patients), but no significant difference was demonstrated (OR 1.85; CI: 0.62, 5.50; p=0.269). Of those who passed away in April 2020, six of the eight patients had been swabbed for COVID-19, and only one of these six tested positive. The patient with a positive COVID swab died of COVID-related chest symptoms.

COVID-19 swab testing

In April 2020, of the 106 patients who required surgical interventions, 47 patients (44%) were tested for the COVID-19 antigen with a nasal swab based their symptoms at or during their admission. The remaining 59 were not tested. Of the 47 patients who were tested for the COVID-19 antigen, only four patients tested positive in our department (8.5% of those who had swabs).

Neck of femur (NOF) sub-group analysis

Forty-nine patients with NOF fractures presented between April 01, 2019 and April 30, 2019 and 49 patients between April 01, 2020 and April 30, 2020. Full datasets were available except for the time in theatre for one operation in April 2019 and three operations in 2020. In April 2019, one patient passed away before surgery, and one patient presented with bilateral NOF fractures which were fixed in the same operation. There was no significant difference between the two subgroups in terms of age, gender, laterality, frequency of subclassification of NOF fractures, nor the frequency of the type of operation. Table 6 illustrates the participant characteristics in full. In April 2020, there were statistically significant increases in spinal anaesthesia from 2% to 38%.

**Table 6:** Participant demographics - neck of femurs (NOF) only

	April 2019	April 2020
Number of patients (n)	49	49
Number of NOFs (n)	50	49
Gender (n)		
Male	16/49	21/49
Female	33/49	28/49
Age (y)		
Median (IQR)	83 (78 – 89)	83 (73 – 87)
Number of operations (n)		
Total	48	49
Mean per day	1.6	1.6
Laterality (n)		
Right	28/49	20/49
Left	20/49	29/49
Bilateral	1/49	0/49
Classification (n)		
Intracapsular	23/49	26/49
Extracapsular	23/49	20/49
Subtrochanteric	3/49	3/49
Operative treatment (n)		
Hemiarthroplasty	17/49	21/49
Total hip replacement	6/49	2/49
Dynamic hip screw	17/49	20/49
Intramedullary nail	7/49	4/49
Cannulated screws	2/49	2/49

There was a slight increase in 30-day mortality in April 2020, but this was not significant (OR 1.47; CI: 0.43, 4.98, p=0.539). Of the seven April 2020 deaths, five were screened with COVID swabs: four of these were negative, and one was positive. The patient with a positive COVID swab died of COVID-related chest symptoms. It should be noted the patients continued to receive shared care with our orthogeriatric colleagues throughout both time periods.

## Discussion

This study shows that even with a national lock down the number of trauma operations only decreased by 30%, but there were no significant changes to the number of hip fractures or polytrauma patients presenting. Even though the operating time, when full PPE protections were implemented for each patient (including not opening theatre doors for 12 minutes after any aerosol-generating procedure), there was only an increase of 14 minutes in total time in theatre. Only 8.5% of patients tested were positive for COVID, which reflected the community exposure at that time, suggesting that trauma patients carry the population risk. Due to the uncertainty of the sensitivity of testing, all patients were treated with full precautions, and whilst there was a small decrease in operative cases of upper limb fractures, all others were treated with standard operative management, without an increase in complications. The negative impact on the training experience of junior surgeons (Core Trainees) should be noted. Whilst this was undoubtedly necessary during the initial surge when hospitals were unprepared, moving forward we need to ensure that the future generations of surgeons are able to meet their training requirements concomitantly. 

When the pandemic was first declared, some institutions reported operations taking two to three times as long to perform, had limited operating capacity, and suggested conservative pathways for hip fractures [6]. Reports from Northern Italy showed that operative treatment of hip fractures in COVID positive patients improved patient stability [7]. NHS England advised continued treatment of hip fractures and surgery should not be delayed in those awaiting test results of positive results [2].

Our study was undertaken during a national lockdown, which prevented all but essential travel and work, and only one hour of outdoor exercise per day. Road traffic trauma and sports-related trauma did decrease as one would expect but falling from a bicycle or horse did increase. The proportional volume of low energy trauma/falls increased.

In future lockdowns or pandemics, hospitals need to continue to provide orthopaedic trauma care, and expect a similar number of hip fracture patients, falls from standing heights, and polytraumas. Our experience in providing this service has not led to a deterioration in outcomes. Limitations of our study are that we did not include the data of patients undergoing non-operative treatment, and times for theatre cleaning were not available. Widespread testing was not available during the study period so the accurate incidence of COVID in the study population was unknown, though the data suggested it represented the population reported incidence, at that time. Strengths of our study are that all trauma patients managed operatively were incorporated and patient numbers were relatively large compared to current literature.

## Conclusions

Our data mandates that orthopaedic trauma services need to be provided during a national lockdown. There was no significant decrease in the volume of patients sustaining falls, which include hip fractures. Mean operating time only increases by 14 minutes with the wearing of full PPE. This should be part of the future planning for any further pandemics or lockdowns.

## Appendices

**Table 7:** List of complications in all trauma patients (Appendix 1)

Complications	April 2019	April 2020
Wound complication	2x stitch abscess 2x wound dehiscence 1x wound necrosis	0
Vascular injury	1x major haemorrhage intra-operatively	1x left profunda artery false aneurysm requiring embolsation 1x obturator artery pseudo-aneurysm requiring embolisation
Nerve injury	1x lateral femoral cutaneous nerve paraesthesia 1x common peroneal nerve injury	1x saphenous nerve paraesthesia
Thromboembolism	7x DVT 2x PE	3x DVT 1x PE
Anaemia requiring transfusion	5	8
Cardiorespiratory	6x hospital-acquired pneumonia 1x ARDS 2x unplanned ICU stay for BP support	4x aspiration pneumonia 1x hospital-acquired pneumonia 1x unplanned ICU stay for BP support
Renal	2x AKI	8x AKI
Other	1x right iliac fossa haematoma	1x erectile dysfunction 1x heterotopic ossification 1x reoperation for mal-reduced syndesmosis 1x anaphylaxis intra-operatively
